# Human papillomavirus detection in liquid-based cytology and cervical histology samples among women living with human immunodeficiency virus in Zambia

**DOI:** 10.1016/j.ijregi.2026.100973

**Published:** 2026-08-11

**Authors:** Katayoun Taghavi, Misinzo Moono, Nathalie V. Fernandez Villalobos, John Andoh, Allison Glass, Partha Basu, Thamsanqa Madliwa, Mulindi H. Mwanahamuntu, Albert Manasyan, Eliane Rohner

**Affiliations:** 1 Institute of Social and Preventive Medicine, University of Bern, Bern, Switzerland; 2 Early Detection, Prevention and Infections Branch, International Agency for Research on Cancer, World Health Organization (IARC/WHO), Lyon, France; 3 Centre for Infectious Disease Research in Zambia, Lusaka, Zambia; 4 Molecular Pathology Department, Lancet Laboratories, Johannesburg, South Africa; 5 Women and Newborn Department, University Teaching Hospital, Lusaka, Zambia; 6 University of Alabama at Birmingham, Birmingham, Alabama, United States of America

**Keywords:** HPV infection, Cytology samples, Histology samples, Women living with HIV

## Abstract

**Objectives:**

Cervicovaginal human papillomavirus (HPV) prevalence is high among women living with human immunodeficiency virus (WLHIV), but not all HPV genotypes detected in liquid-based cytology (LBC) may be present in cervical tissue. We explored HPV genotype concordance in paired LBC and histology samples among WLHIV in Zambia.

**Methods:**

WLHIV aged 18-65 years who were enrolled in a cervical screening test accuracy study were invited for follow-up after 30-36 months. LBC samples and two or more cervical biopsies were collected and tested for 28 HPV genotypes (Anyplex II/Allplex HPV28, Seegene, South Korea). HPV prevalence and genotype-specific agreement were assessed using Cohen’s kappa.

**Results:**

From April 2022 to May 2023, 242 WLHIV (22 with high-grade squamous intraepithelial lesions) had valid paired HPV results. The prevalence of any HPV was similar on LBC (39%) and histology (34%). Multiple-type infections were more common on LBC (18%) than on histology (10%). HPV 16 was the most frequent genotype: 9% (n = 22) on LBC, and 15% (n = 36) on histology. Agreement for any high-risk HPV genotype was 66.1% (kappa = 0.27).

**Conclusions:**

Among Zambian WLHIV with mostly low-grade cervical lesions, LBC showed more multiple-type HPV infections and limited agreement with histology samples, possibly reflecting transient infections detected by LBC. The frequent detection of HPV 16 in histology samples but not in LBC warrants further investigation.

## Highlights

• High-risk human papillomavirus (HPV) prevalence was similar in cytology (39%) and histology (34%) samples among women living with human immunodeficiency virus.
• Multiple HPV infections were nearly twice as common in cytology than in histology samples.
• Agreement for any HPV genotype between cytology and histology was fair (κ = 0.27).
• HPV 16 was more frequently detected in histology than cytology samples.

## Introduction

Persistent cervicovaginal infection with human papillomavirus (HPV) is a necessary cause of cervical intraepithelial neoplasia (CIN), which can progress to cervical cancer. Although hundreds of HPV genotypes have been identified, only a small subset is associated with cancer [1,2]. The International Agency for Research on Cancer (IARC) classifies HPV genotypes 16, 18, 31, 33, 35, 39, 45, 51, 52, 56, 58, and 59 as group 1 carcinogens, with sufficient evidence of their causal role in cervical cancer [3]. HPV 68 is classified as group 2A (probably carcinogenic to humans), whereas HPV genotypes 26, 30, 34, 53, 66, 67, 69, 70, 73, 82, 85, and 97 are classified as group 2B (possibly carcinogenic), based on limited evidence or phylogenetic similarity to known carcinogenic genotypes [3]. In a large pooled analysis, women with cervical cancer had nearly 50 times higher odds of being infected with HPV 16 and 13 times higher odds of being infected with HPV 18 than women with normal cytology [4]. The associations of other high-risk HPV genotypes with cervical cancer were weaker but still clinically relevant.

Knowledge of cervical cancer risk associated with HPV genotypes is increasingly used for developing risk-based cervical screening and treatment strategies. However, effective application requires understanding the concordance and clinical relevance of HPV genotypes detected across different sample types. Previous studies among women who were HIV-negative that compared HPV genotype detection in liquid-based cytology (LBC) and histology samples generally reported high but not perfect agreement [[5], [6], [7], [8], [9], [10], [11], [12], [13], [14]], with discrepancies often attributed to biological differences in the sampling methods. LBC samples capture exfoliated cells from across the cervical transformation zone, whereas histology samples—usually obtained after a detailed colposcopy examination of the cervix—often reflect small, targeted areas of tissue. HPV infections detected in LBC samples can represent either persistent or transient infections, including infections that have not yet caused morphologic or neoplastic abnormalities [15]. In contrast, HPV genotypes detected in histology samples may reflect tissue-level evidence of HPV infection.

Women living with HIV (WLHIV) have a higher prevalence of cervicovaginal HPV infection than their HIV-negative counterparts [16], [17], [18]. Because many of these HPV infections are transient, and often involve multiple genotypes, HPV test specificity among WLHIV is impaired, limiting the usefulness of a positive HPV test alone for clinical decision-making in this population [17], [18], [19]. More information is needed to better understand the degree to which LBC-detected genotypes correspond with those involved in histologic lesions and the biological significance of concordant and discordant HPV detection between these sample types [19]. Therefore, we assessed HPV genotype agreement in paired cervical LBC and histology samples in a cohort of WLHIV attending cervical screening in Lusaka, Zambia.

## Methods

### Study design and population

We included WLHIV aged 18-65 years who participated in a cervical screening test accuracy study between May 2019 and March 2021 and returned for follow-up after 30-36 months. We excluded women if they had a history of cervical cancer, had undergone hysterectomy, or were pregnant. The study was conducted at Kanyama Hospital in Lusaka, Zambia; methods and results of the underlying test accuracy study have been described in detail elsewhere [20]. At baseline, most women had never been screened and were on antiretroviral therapy (ART). We attempted to re-contact all participants in the screening test accuracy study and invited them to attend a 30-36 month follow-up visit. The present analysis used paired HPV test results from the 30-36 month follow-up visit and included all participants who returned for the follow-up visit and had valid paired HPV test results available.

### Study procedures

All participants had a speculum examination, and a trained nurse collected an LBC sample using cervix brushes (Rovers, The Netherlands). After collection, the cervix brush was placed into ThinPrep PreservCyt solution (Hologic, USA). This was followed by a colposcopic examination using the Gynocular colposcope (Gynius, Sweden). Between two and four cervical biopsies were obtained from each participant. If cervical lesions were observed, biopsies were taken from the two most severe areas or more. If no visible lesions were present, biopsies were systematically obtained from all four quadrants of the transformation zone.

The LBC and biopsy samples were sent to the Lancet Laboratory in Pretoria, South Africa for HPV testing using the Anyplex II HPV28 HR detection system (Seegene, South Korea). In April 2023, Anyplex production was discontinued and replaced by Allplex (Seegene, South Korea). HPV testing on cervical histology specimens was conducted using formalin-fixed paraffin-embedded (FFPE) tissue blocks. Tissue sections were cut, stained with hematoxylin and eosin, and examined under light microscopy by an accredited pathologist. To minimize cross-contamination between specimens, a new microtome blade was used for each tissue block, and the microtome was cleaned between samples. Sections containing a high proportion of histologically abnormal epithelium were identified and used for DNA extraction. Deparaffinization was performed by incubating tissue sections in a buffer–proteinase solution at 70°C for 2 hours. After enzymatic digestion, lysis buffer was added, and the samples were vortexed. DNA extraction followed the FFPE Plus DNA protocol, using the Maxwell RSC 16 automated system (Promega, USA). HPV DNA detection was carried out using the Anyplex II/Allplex HPV28 assay. This assay has been formally validated for cervical swab specimens and LBC samples. Although FFPE tissue specimens are not included in the manufacturer’s validated specimen types, the use of FFPE-derived DNA for HPV detection with PCR-based assays, including the Anyplex platform, has been reported in the published literature [14]. The analytic platforms were used by trained staff who underwent regular quality assurance monitoring. Repeat testing or formal reproducibility assessment was not performed. Histopathologic samples were assessed by expert pathologists using the World Health Organization (WHO) Classification of Tumours of Female Reproductive Organs, including p16 immunostaining [21]. Low-grade squamous intraepithelial lesion was defined as CIN1 or p16-negative CIN2, showing HPV-associated changes confined to the lower third of the epithelium, with koilocytic atypia. High-grade squamous intraepithelial lesion (HSIL) included CIN2 and CIN3, classified as HSIL only when CIN2 was p16-positive and characterized by atypical squamous cells extending into the middle or upper third of the epithelium.

Study data, including test results, were recorded using paper case report forms. A trained data associate entered data from paper case report forms into a password-protected electronic database (Research Electronic Data Capture, REDCap, Vanderbilt University, USA).

### Statistical analysis

HPV genotypes were grouped according to their carcinogenic classification based on IARC Monograph evaluations and genotype groupings used by the assay manufacturer. The group 1 (carcinogenic to humans) genotypes included HPV 16, 18, 31, 33, 35, 39, 45, 51, 52, 56, 58, and 59. Group 2 included HPV 68 (2A, probably carcinogenic) and 26, 53, 66, 69, 73, and 82 (group 2B, possibly carcinogenic). Not all IARC group 2B HPV genotypes are detected by the Anyplex II/Allplex HPV28 assay; therefore, only a subset of group 2B genotypes is represented in this category. The low-risk HPV genotypes included HPV 6, 11, 40, 42, 43, 44, 54, 61, and 70. We also defined an “any high-risk HPV” category that included all group 1, group 2A, and group 2B genotypes. We defined cervical disease as the detection of HSIL on histologic assessment.

We estimated HPV prevalence in LBC and histology samples, with corresponding 95% confidence intervals (CIs), for individual group 1 HPV genotypes and different HPV risk groups. We determined the proportion of single vs multiple-type HPV infections by specimen type. We evaluated the overall and positive agreement between HPV detection in LBC and histology samples. We defined positive agreement for individual group 1 genotypes and different HPV risk groups as *2a/(2a + b + c)*, where *a* represents the number of women who tested positive on both sample types, and *b* and *c* represent the number of women who tested positive on one sample type only. We assessed agreement beyond chance using Cohen’s kappa coefficient, with 95% CIs, for individual group 1 HPV genotypes and different HPV risk groups. To assess whether HPV detection differed systematically between paired LBC and histology samples, we used the exact McNemar’s test for paired proportions. We report analyses of agreement beyond chance overall and stratified by cervical disease status (HSIL vs <HSIL) or precancer treatment received between the baseline and follow-up visit (yes vs no). Kappa values were interpreted using the following thresholds: ≤0 (no agreement), 0.01-0.20 (slight), 0.21-0.40 (fair), 0.41-0.60 (moderate), 0.61-0.80 (substantial), and 0.81-1.00 (almost perfect agreement). CIs for the kappa statistic were estimated via nonparametric bootstrap resampling with 1000 iterations, where the dataset was resampled with replacement, and kappa was recalculated in each iteration.

## Results

### Study population

Of 375 women who participated in the initial screening test accuracy study, 247 (66%) returned for the 30-36-month follow-up visit (Supplementary Figure 1). We excluded five women with missing HPV genotype results on either LBC or histology at follow-up, leaving 242 participants with valid paired HPV test results. Of those, 241 had valid histopathology results, and 21 (8.7%) had HSIL. The median age at follow-up was 40.6 years (interquartile range: 35.1-47.2) (Table 1). At baseline, most women were in a relationship or married (69.0%) and had no secondary school education (51.8%), and 60.3% reported no income in the preceding 3 months. All women were on ART, and their median CD4 cell count was 736 cells/mm³ (interquartile range: 576-951).

**Table 1:** Participant characteristics.

Variables	Study population (n = 242)
**Age (years)**	
Median (IQR)	40.6 (35.1 to 47.2)
**Marital status**^a^**, n (%)**	
Single	7 (2.9%)
In a relationship/Married	167 (69.0%)
Separated/ divorced/widowed	68 (28.1%)
**Highest level of education**^a^**, n (%)**	
None	27 (11.2%)
Primary (started/finished)	98 (40.6%)
Secondary (started/finished)	109 (45.6%)
Tertiary (college/university/vocational)	8 (3.4%)
**Income in ZMW**^a^**^,^**^b^**, n (%)**	
No income	146 (60.3%)
Any income	96 (39.7%)
**Smoking**^a^**, n (%)**	
Ever smoked	7 (2.9%)
Never smoked	235 (97.1%)
**Age at sexual debut**^a^**(years)**	
Median (IQR)	17 (16-19)
**Contraception**^a^**, n (%)**	
Yes	93 (38.4%)
*COC pill*	*10 (10.7%)*
*Condoms*	*21 (22.6%)*
*Depot Provera*	*45 (48.4%)*
*Other*	*17 (18.3%)*
No	149 (61.6%)
**Parity**^a^	
Median (IQR)	4 (2-5)
**Tested positive for HPV at baseline visit**^c^	
Yes	106 (43.8%)
No	135 (55.8%)
Missing	1 (0.4%)
**History of treatment for cervical precancer before baseline visit**	
Yes	5 (2.1%)
No	237 (97.9%)
**Treatment for cervical precancer after baseline study visit**	
Yes	55 (22.7%)
No	185 (76.5%)
Missing	2 (0.8%)
**On ART**^a^**, n (%)**	
Yes	242 (100.0%)
No	0
**CD4 cell count (cells/mm^3^)**	
Median (IQR)	736 (576 to 951)
**HIV RNA viral load (copies/mL)**	
Undetectable (<30 copies/mL)	208 (86.0%)
Detectable	34 (14.0%)

1 ZMW = USD $0.04; ART, antiretroviral therapy; CD4, cluster of differentiation 4 T lymphocyte; COC pill, combined oral contraceptive pill; Depot Provera, depot medroxyprogesterone acetate (an injectable contraceptive); HIV, human immunodeficiency virus; HPV, human papillomavirus; IQR, interquartile range; ZMW, Zambian Kwacha.

a Based on data from the baseline study visit.

b Income is reported for the 3 months preceding enrollment.

c At baseline, GeneXpert alone was used for HPV testing; at follow-up, GeneXpert and Anyplex/Allplex testing platforms were used.

### HPV prevalence

The prevalence of any hrHPV genotype was 39.2% (n = 95 of 242) in LBC samples and 34.3% (n = 83 of 242) in histology samples (*P* = 0.224). Multiple infections (including low- and high-risk HPV genotypes) were nearly twice as common in LBC samples (18.2%, n = 44 of 242) as in histology samples (10.3%, n = 25 of 242). For nine (3.7%) women, multiple HPV infections were detected in LBC and histology samples. Genotype-specific HPV prevalence was generally higher in LBC samples than in histology samples (Figure 1), except for HPV 16, which had a prevalence of 9.1% (95% CI: 6.1-13.5%, n = 22 of 242) in LBC samples and 14.9% (95% CI: 10.9-20.0%, n = 36 of 242) in histology samples (*P* = 0.039). Among women with HSIL, HPV 16 detection was 38.1% (n = 8 of 21) in LBC samples and 23.8% (n = 5 of 21) in histology samples. In contrast, among women with <HSIL, HPV 16 detection was less frequent in LBC samples (6.4%, n = 14 of 220) than in histology samples (14.1%, n = 31 of 220). All other individual group 1 HPV genotypes were detected in less than 10% of either LBC or histology samples.

**Figure 1:**
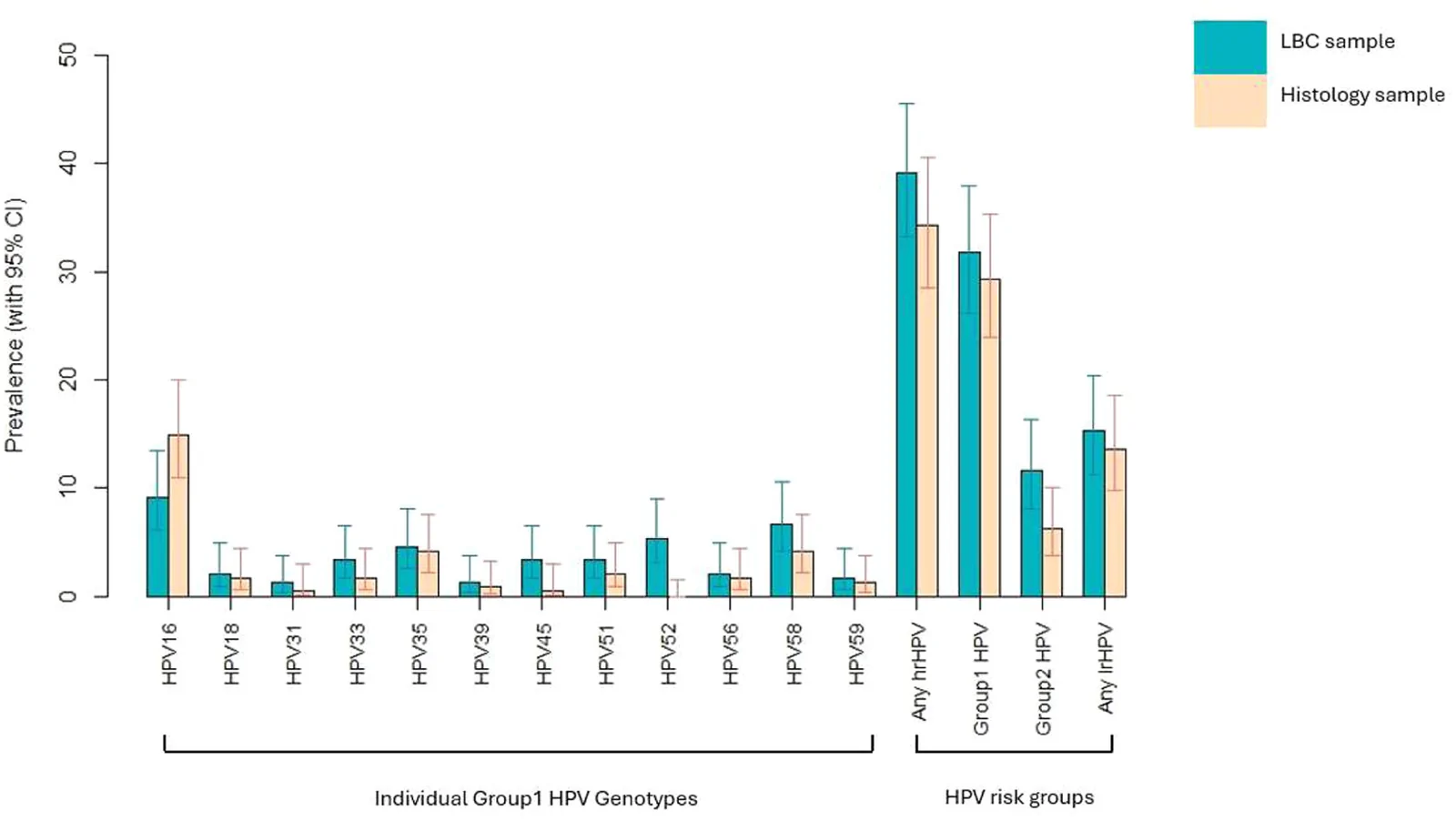
HPV prevalence in liquid-based cytology (LBC) and histology samples. CI, confidence interval; HPV, human papillomavirus; hrHPV, high-risk HPV genotype; LBC, liquid-based cytology; lrHPV, low-risk HPV. Group 1 HPV: any International Agency for Research on Cancer group 1 carcinogenic genotypes (16, 18, 31, 33, 35, 39, 45, 51, 52, 56, 58, 59). Group 2 HPV: any International Agency for Research on Cancer group 2 genotypes (2A probably carcinogenic: 68; 2B possibly carcinogenic: 26, 53, 66, 69, 73, 82).

Figure 2 shows the distribution of HPV positivity by sample type. Among the 130 women who tested positive for any high-risk HPV genotype in at least one sample type, 48 (36.9%) women tested positive on LBC and histology. Among the individual group 1 genotypes, HPV 58 had the highest proportion of positive tests on LBC and histology (52.9%, n = 9 of 17), whereas the proportion was substantially lower for HPV 16 (18.4%, n = 9 of 49). Notably, HPV 31, HPV 52, and HPV 59 were never detected in both sample types. The proportion detected in both sample types was higher for any high-risk HPV genotype (36.9%, n = 48 of 130) than for low-risk HPV (16.7%, n = 10 of 60).

**Figure 2:**
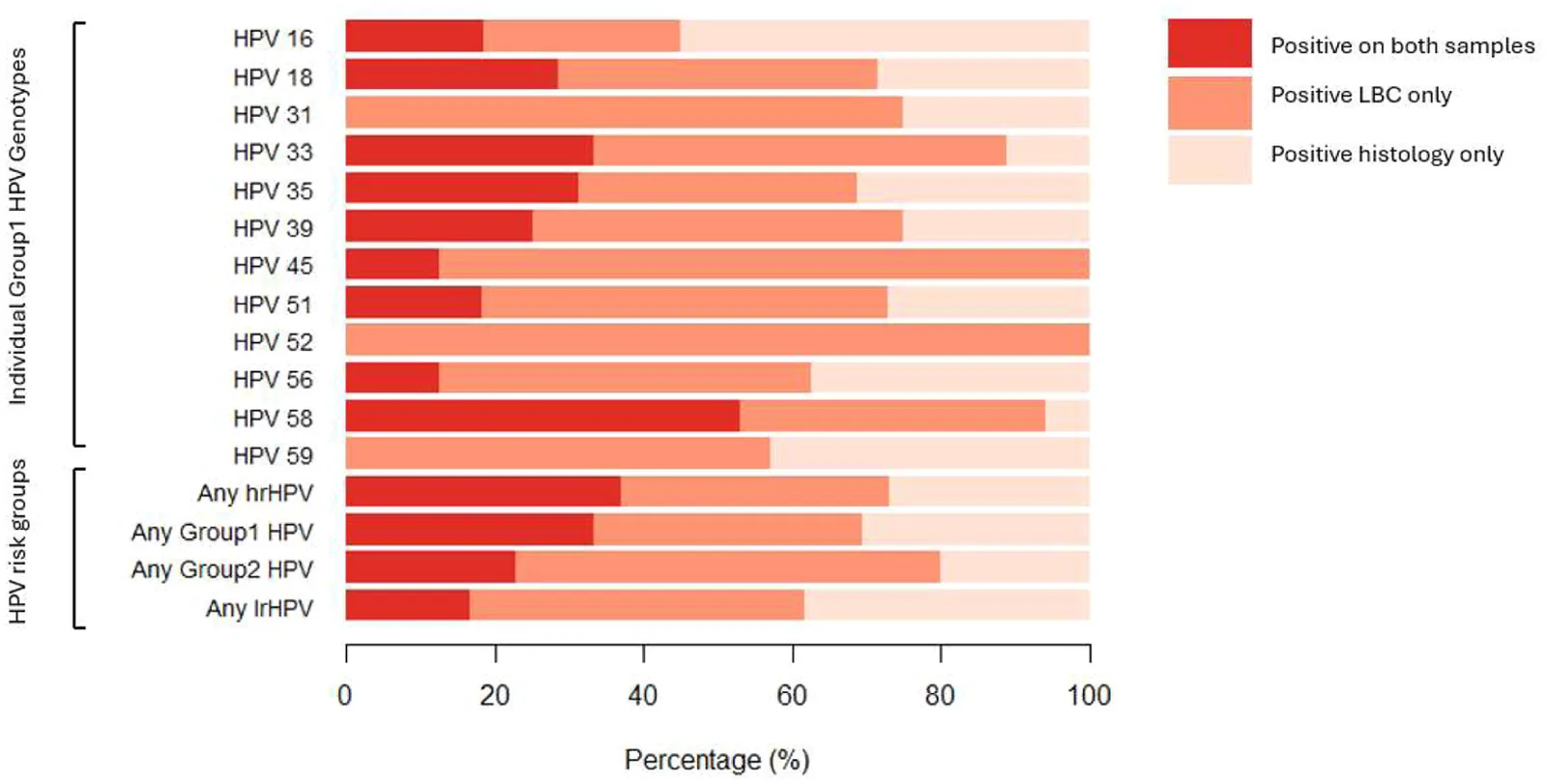
Distribution of HPV positivity by sample type. HPV, human papillomavirus; hrHPV, high-risk HPV genotype; LBC, liquid-based cytology; lrHPV, low-risk HPV. Group 1 HPV: any International Agency for Research on Cancer group 1 carcinogenic genotypes (16, 18, 31, 33, 35, 39, 45, 51, 52, 56, 58, 59). Group 2 HPV: any International Agency for Research on Cancer group 2 genotypes (2A probably carcinogenic: 68; 2B possibly carcinogenic: 26, 53, 66, 69, 73, 82).

### Agreement

For individual group 1 HPV genotypes, the overall percentage agreement between detection in LBC and histology samples was generally high, ranging from 83.5% for HPV 16 to 98.7% for HPV 39, but this largely reflected the high proportion of women who tested negative on both sample types. For the “any high-risk HPV genotype” category, overall agreement was lower, at 66.1% (Table 2). Positive agreement, which reflects concordance among women with HPV detected on at least one specimen type, was substantially lower. For most individual group 1 genotypes, positive agreement was ≤50.0%, except for HPV 58, which had a positive agreement of 69.2%. Accordingly, Cohen’s kappa indicated low to moderate agreement beyond chance for individual group 1 genotypes. For example, positive agreement for HPV 16 was 31.0%, and agreement beyond chance was fair (kappa 0.22, 95% CI: 0.06-0.39). Several genotypes, including HPV 31, 52, and 59, demonstrated no positive agreement. Individual group 2 and low-risk HPV genotypes were infrequently detected, with most genotypes, except for HPV 53, HPV 42, and HPV 70, showing fair agreement beyond chance or less (Supplementary Table 1). Positive agreement was generally higher among women with HSIL than among those with <HSIL, except for any group 2 HPV (Table 3). For HPV 16, positive agreement was 61.5% among women with HSIL and 22.2% among women with <HSIL. Some agreement estimates were also higher among women who had received precancer treatment (n = 46) than among those who had not (n = 196), particularly for HPV 16 and any high-risk HPV genotype (Supplementary Table 2). However, the stratified analyses should be interpreted cautiously due to the small sample size.

**Table 2:** Agreement between cervical cytology and histology samples for individual group 1 HPV genotypes and HPV risk groups.

			Histology		Total	Overall agreement (%)	Positive agreement (%)	Kappa (95% CI)
	HPV result	Cytology	Positive	Negative				
Individual Group 1 HPV genotypes	HPV 16	Positive	9	13	22			0.22
		Negative	27	193	220	83.5	31.0	(0.06 to 0.39)
		Total	36	206	242			
	HPV 18	Positive	2	3	5			0.43
		Negative	2	235	237	97.9	44.4	(0.02 to 0.84)
		Total	4	238	242			
	HPV 45	Positive	1	7	8			0.22
		Negative	0	234	234	97.1	22.2	(−0.14 to 0.57)
		Total	1	241	242			
	HPV 31	Positive	0	3	239			−0.01
		Negative	1	238	3	98.3	0	(−0.02 to 0.01)
		Total	1	241	242			
	HPV 33	Positive	3	5	8			−0.01
		Negative	1	233	234	97.5	50.0	(−0.03 to 0.00)
		Total	4	238	242			
	HPV 35	Positive	5	6	11			0.45
		Negative	5	226	231	95.5	47.6	(0.18 to 0.72)
		Total	10	232	242			
	HPV 52	Positive	0	13	13			0.00
		Negative	0	229	229	94.6	0	(not estimable)
		Total	0	242	242			
	HPV 58	Positive	9	7	16			0.68
		Negative	1	225	226	96.7	69.2	(0.47 to 0.89)
		Total	10	232	242			
	HPV 39	Positive	1	2	3			0.39
		Negative	1	238	239	98.7	40.0	(−0.15 to 0.94)
		Total	2	240	242			
	HPV 59	Positive	0	4	4			−0.01
		Negative	3	235	238	97.1	0	(−0.03 to 0.00)
		Total	3	239	242			
	HPV 51	Positive	2	6	8			0.29
		Negative	3	231	234	96.2	30.8	(−0.04 to 0.62)
		Total	5	237	242			
	HPV 56	Positive	1	4	5			0.21
		Negative	3	234	237	97.1	22.2	(−0.16 to 0.57)
		Total	4	238	242			
HPV risk groups	Any hrHPV	Positive	48	47	95			0.27
	(all 19 genotypes)	Negative	35	112	147	66.1	54.0.	(0.15 to 0.40)
		Total	83	159	242			
	Any group 1	Positive	37	40	77			0.28
	(12 genotypes)	Negative	34	131	165	69.4	50.0	(0.15 to 0.41)
		Total	71	171	242			
	Any group 2	Positive	10	23	33			0.33
	(7 genotypes)	Negative	8	201	209	87.1	39.2	(0.15 to 0.51)
		Total	18	224	242			
	Any lrHPV	Positive	10	27	37			0.17
	(9 genotypes)	Negative	23	182	205	79.3	28.6	(0.01 to 0.32)
		Total	33	209	242			

Kappa, Cohen’s kappa; CI, confidence interval; HPV, human papillomavirus; hrHPV, high-risk HPV genotype; LBC, liquid-based cytology; lrHPV, low-risk HPV.

Group 1 HPV: International Agency for Research on Cancer group 1 carcinogenic genotypes (16, 18, 31, 33, 35, 39, 45, 51, 52, 56, 58, 59).

Group 2 HPV: International Agency for Research on Cancer group 2 genotypes (2A probably carcinogenic: 68; 2B possibly carcinogenic: 26, 53, 66, 69, 73, 82).

**Table 3:** Agreement of HPV types in cervical cytology and histology samples among women with HSIL (n = 21) and <HSIL (n = 220)^a^.

HPV result	Cytology	Histology			HSIL		
		Positive	Negative	Total	Overall agreement (%)	Positive agreement (%)	Kappa (95% CI)
HPV 16	Positive	4	4	8	76.2	61.5	0.46
	Negative	1	12	13			(0.07 to 0.84)
	Total	5	16	21			
Any hrHPV	Positive	16	4	20	76.2	86.5	−0.08
(all 19 genotypes)	Negative	1	0	1			(−0.24 to 0.08)
	Total	17	4	21			
Any group 1	Positive	2	1	3	76.2	84.8	0.31
(12 genotypes)	Negative	4	14	18			(0.13 to 0.76)
	Total	6	15	21			
Any group 2	Positive	2	2	4	76.2	44.4	0.29
(7 genotypes)	Negative	3	14	17			(−0.18 to 0.77)
	Total	5	16	21			
Any lrHPV	Positive	1	1	2	85.7	40.0	0.32
(9 genotypes)	Negative	2	17	19			(−0.26 to 0.91)
	Total	3	18	21			


HPV result	Cytology	Histology			<HSIL		
		Positive	Negative		Overall agreement (%)	Positive agreement (%)	Kappa (95% CI)
HPV 16	Positive	5	9	14	84.1	22.2	0.15
	Negative	26	180	206			(−0.02 to 0.31)
	Total	31	189	220			
Any hrHPV	Positive	32	42	74	65.5	45.7	0.21
(all 19 genotypes)	Negative	34	112	146			(0.07 to 0.34)
	Total	66	154	220			
Any group 1	Positive	23	35	58	63.6	40.4	0.19
(12 genotypes)	Negative	33	129	162			(−0.05 to 0.33)
	Total	56	164	220			
Any group 2	Positive	8	21	29	88.2	38.1	0.32
(7 genotypes)	Negative	5	186	191			(0.13 to 0.52)
	Total	13	207	220			
Any lrHPV	Positive	9	26	35	78.6	27.6	0.15
(9 genotypes)	Negative	21	164	185			(−0.01 to 0.31)
	Total	30	190	220			

kappa, Cohen’s kappa; CI, confidence interval; HPV, human papillomavirus; hrHPV, high-risk HPV genotype; HSIL, high-grade squamous intraepithelial lesion; lrHPV, low-risk HPV.

Group 1 HPV: International Agency for Research on Cancer group 1 carcinogenic genotypes (16, 18, 31, 33, 35, 39, 45, 51, 52, 56, 58, 59).

Group 2 HPV: International Agency for Research on Cancer group 2 genotypes (2A probably carcinogenic: 68; 2B possibly carcinogenic: 26, 53, 66, 69, 73, 82).

a A total of 241 because of one missing histology for grade of disease.

## Discussion

In our study among WLHIV in Zambia, we found that hrHPV infections were detected more frequently in LBC than in histology samples, and multiple HPV infections were twice as common in LBC as in histology samples. The overall agreement for the “any HPV genotype” category between LBC and histology was high, but this was largely driven by the high proportion of women who tested negative on both sample types. In contrast, positive agreement, which better reflects concordance among women who tested positive for HPV, was considerably lower for most genotypes. In the HPV genotype-specific analyses, positive agreement was often ≤50%, and kappa ranged from no agreement to moderate agreement beyond chance. HPV 16 was the most frequently detected individual genotype and was more often identified in histology samples than in LBC samples, suggesting differences in infection biology and sampling.

To the best of our knowledge, our study is one of the few to examine agreement between HPV detection in LBC and histology samples among WLHIV in sub-Saharan Africa. We analyzed paired LBC and histology samples collected at the same study visit, minimizing temporal misclassification. Sample handling and DNA extraction procedures were standardized in a single reference laboratory, with defined FFPE extraction and quality assurance procedures. We used a single HPV genotyping platform for both samples, avoiding inter-assay variability; however, it is acknowledged that the Anyplex II/Allplex HPV28 assays have not been formally validated for use on FFPE specimens. We reduced detection and misclassification bias by obtaining two to four biopsies from each woman and classifying cervical disease as HSIL on histology. Our analysis expanded beyond published studies that focused on overall agreement [5,9,12] by reporting positive agreement as well as genotype-specific concordance and Cohen’s kappa. However, several limitations need to be considered. Kappa can be attenuated when the outcome prevalence is low. Given the low prevalence of certain HPV genotypes, kappa should be interpreted cautiously and in combination with the overall and, in particular, positive agreement, because overall agreement may also be inflated by the large number of negative–negative pairs [[22], [23], [24]]. Although contamination during tissue sectioning is a recognized concern in FFPE-based HPV studies, standard laboratory procedures were used to minimize this risk, including the use of a new microtome blade for each specimen and thorough cleaning of the microtome between blocks. Nevertheless, differences in HPV detection may reflect variation in DNA yield between specimen types, sampling variability, tissue heterogeneity, or limitations of FFPE-derived DNA. The generalizability of our results may also be limited because most WLHIV included in our study were on ART and virologically well controlled. HIV-induced immunosuppression is associated with a higher HPV prevalence and more multiple-type infections, which may differentially affect HPV detection in LBC and histology samples [25]. We lacked biomarkers such as E6/E7 mRNA or p16/Ki-67 dual staining, which indicates viral oncogene activity and host cell transformation. This information could have helped to identify progressive lesions, even if histologic neoplasia was not yet evident, clarifying the clinical significance of histology-only HPV 16 among women without HSIL. Lastly, our sample size was small, particularly among women with HSIL, limiting the precision of some genotype-specific estimates. These findings should, therefore, be interpreted cautiously and require confirmation in larger studies.

In line with our results, studies conducted among women of the general population in Europe, America, and Asia also reported higher hrHPV prevalence and more frequent multiple-type infections in LBC than in matched histology samples [[24], [25], [26], [27], [28], [29], [30]]. This is likely due to the sampling of the whole transformation zone with LBC, which may capture more transient HPV infections that could be missed by colposcopy-directed biopsies [[5], [6], [7], [8], [9], [10],^19^]. One study among Kenyan WLHIV reported a similar pattern of higher HPV detection and more frequent multiple-type infections in LBC than in histology samples, with agreement improving with lesion severity [19]. A Thai study among men living with HIV who have sex with men assessed HPV-related anal disease and found a higher prevalence of any hrHPV genotype and HPV 16 in anal LBC than in histology samples [25]. In contrast to other genotypes, we found that HPV 16 was more frequently detected in histology samples than in LBC samples. This finding was primarily driven by the higher proportions of HPV 16 detected in histology samples than in LBC samples among women with <HSIL. Among women with HSIL, HPV 16 detection was higher in LBC than in histology samples. Given the small number of women with HSIL, these findings should be interpreted cautiously. Nevertheless, sampling differences may also have contributed to this observation, with LBC capturing infections in exfoliated cells from across the cervix and histology samples reflecting HPV genotypes present within specific tissue samples. However, it is unexpected to see HPV 16 detected less frequently in histology samples than in LBC among women with HSIL because cervical biopsies per protocol were directed toward colposcopically suspected HSIL lesions, where higher HPV 16 detection in histology samples would be anticipated. If there is less cellular shedding in <HSIL tissue than in HSIL tissue, this could result in higher detection of HPV 16 in histology samples than in LBC samples, even before HPV infection has developed into tissue neoplasia, but this has not been reported in other studies. Assessing biomarkers (e.g. E6/E7 mRNA, p16/Ki-67) could help clarify whether these histology-only HPV 16 detections reflect transforming, clinically relevant cervical disease.

The 2021 WHO cervical cancer screening guidelines recommend HPV detection tests, typically done on LBC samples, as the primary screening method for all women, including WLHIV [18]. However, it is noted that among WLHIV, HPV testing on LBC samples has limited specificity [18]. Therefore, the WHO recommends the use of a triage test before treatment decisions are made in WLHIV who test positive for hrHPV [18]. In WLHIV, LBC samples often capture transient or multiple-type infections, which may not necessarily reflect the genotype that drives cervical lesion development [26,27]. In our study, multiple-type infections were nearly twice as common in LBC samples compared with histology samples. The higher detection of HPV 16 in histology samples warrants further investigation. Moreover, additional research is needed to determine the extent to which HPV genotypes detected in LBC correspond to those present within cervical tissue and the biological significance of any differences, particularly among WLHIV. Future studies should also prospectively follow WLHIV with paired LBC and biopsy samples and incorporate biomarkers of oncogenic transformation, such as E6/E7 mRNA, p16/Ki-67 dual staining, or host and viral DNA methylation, to clarify whether HPV genotypes identified in tissue more frequently represent persistent infections that may progress to HSIL or cancer than HPV genotypes detected in LBC.

Among previously screened WLHIV in Zambia, we found a higher HPV prevalence and increased frequency of multiple-type infections in LBC than histology samples. HPV genotype concordance between the sample types was limited. A substantial subset of women had HPV 16 detected in histology samples only, suggesting that HPV 16 detection may differ according to specimen type. The clinical significance of HPV 16 detected on cervical histology only, particularly among women without HSIL, warrants further investigation.

## CRediT authorship contribution statement

**Katayoun Taghavi:** Conceptualization, Data curation, Formal analysis, Funding acquisition, Investigation, Methodology, Project administration, Visualization, Writing – original draft, Writing – review & editing. **Misinzo Moono:** Data curation, Investigation, Project administration, Supervision, Validation, Writing – review & editing. **Nathalie V. Fernandez Villalobos:** Data curation, Visualization, Writing – review & editing. **John Andoh:** Data curation, Investigation, Writing – review & editing. **Allison Glass:** Data curation, Investigation, Writing – review & editing. **Partha Basu:** Conceptualization, Funding acquisition, Methodology, Resources, Supervision, Writing – review & editing. **Thamsanqa Madliwa:** Data curation, Investigation, Validation, Writing – review & editing. **Mulindi H. Mwanahamuntu:** Conceptualization, Investigation, Methodology, Resources, Supervision, Writing – review & editing. **Albert Manasyan:** Conceptualization, Funding acquisition, Investigation, Methodology, Resources, Writing – review & editing. **Eliane Rohner:** Conceptualization, Data curation, Funding acquisition, Methodology, Supervision, Writing – review & editing.

## Declaration of competing interest

The authors have no competing interests to declare.

## Appendix Supplementary materials

Image, application 1

## References

[1] Muñoz N., Bosch F.X., de Sanjosé S., Herrero R., Castellsagué X., Shah K.V. (2003). Epidemiologic classification of human papillomavirus types associated with cervical cancer. N Engl J Med.

[2] Clifford GM. (2024). Expanding the list of cancers recognized to be caused by infectious agents. J Natl Cancer Inst.

[3] (2012). Biological agents. IARC Monogr Eval Carcinog Risks Hum.

[4] Wei F., Georges D., Man I., Baussano I., Clifford GM. (2024). Causal attribution of human papillomavirus genotypes to invasive cervical cancer worldwide: a systematic analysis of the global literature. Lancet.

[5] Castro F.A., Koshiol J., Quint W., Wheeler C.M., Gillison M.L., Vaughan L.M. (2015). Detection of HPV DNA in paraffin-embedded cervical samples: a comparison of four genotyping methods. BMC Infect Dis.

[6] Lagheden C., Eklund C., Kleppe S.N., Unger E.R., Dillner J., Sundström K. (2016). Validation of a standardized extraction method for formalin-fixed paraffin-embedded tissue samples. J Clin Virol.

[7] Tardío J.C., Cambero O., Sánchez-Estévez C., Sánchez-García A.B., Angulo F., Moreno A. (2019). High-risk human papillomavirus determination in formalin-fixed, paraffin-embedded cervical tissue using the Roche Cobas 4800 system: a comparative study with liquid-based cytology. Int J Gynecol Pathol.

[8] Torii Y., Fujii T., Kukimoto I., Saito M., Iwata T., Takahashi H. (2016). Comparison of methods using paraffin-embedded tissues and exfoliated cervical cells to evaluate human papillomavirus genotype attribution. Cancer Sci.

[9] Dal Bello B., Spinillo A., Alberizzi P., Cesari S., Gardella B., Silini E.M. (2009). Validation of the SPF10 LiPA human papillomavirus typing assay using formalin-fixed paraffin-embedded cervical biopsy samples. J Clin Microbiol.

[10] Kim G., Cho H., Lee D., Park S., Lee J., Wang H.Y. (2017). Comparison of FFPE histological versus LBP cytological samples for HPV detection and typing in cervical cancer. Exp Mol Pathol.

[11] Alberizzi P., Spinillo A., Gardella B., Cesari S., Silini EM. (2014). Evaluation of the HPV typing INNO-LiPA EXTRA assay on formalin-fixed paraffin-embedded cervical biopsy samples. J Clin Virol.

[12] Harlé A., Guillet J., Thomas J., Sastre-Garau X., Rouyer M., Ramacci C. (2018). Evaluation and validation of HPV real-time PCR assay for the detection of HPV DNA in oral cytobrush and FFPE samples. Sci Rep.

[13] Witt C.E., Sutton E.F., Stansbury A.M., Winters A.N., Konur L.C., Luo M. (2024). Characterization of high-risk-other human papillomavirus genotypes in Papanicolaou tests, high-grade squamous intraepithelial lesions, and cervical cancer. Ochsner J.

[14] Almeida M., Caeiro V., Costa D., Silva L., Sousa C., Pestana P. (2024). High-risk HPV detection in paraffin-embedded tissue from cervical lesions. Pharmaceuticals (Basel).

[15] Gravitt P.E., van Doorn L.J., Quint W., Schiffman M., Hildesheim A., Glass A.G. (2007). Human papillomavirus (HPV) genotyping using paired exfoliated cervicovaginal cells and paraffin-embedded tissues to highlight difficulties in attributing HPV types to specific lesions. J Clin Microbiol.

[16] Palefsky J.M., Minkoff H., Kalish L.A., Levine A., Sacks H.S., Garcia P. (1999). Cervicovaginal human papillomavirus infection in human immunodeficiency virus-1 (HIV)-positive and high-risk HIV-negative women. J Natl Cancer Inst.

[17] Stelzle D., Tanaka L.F., Lee K.K., Ibrahim Khalil A., Baussano I., Shah A.S.V. (2021). Estimates of the global burden of cervical cancer associated with HIV. Lancet Glob Health.

[18] World Health Organization (WHO). WHO guideline for screening and treatment of cervical pre-cancer lesions for cervical cancer prevention. 2nd ed. *Geneva*, 2021. https://www.who.int/publications/i/item/9789240040434. [accessed 8 January 2026]. 34314129

[19] De Vuyst H., Chung M.H., Baussano I., Mugo N.R., Tenet V., van Kemenade F.J. (2013). Comparison of HPV DNA testing in cervical exfoliated cells and tissue biopsies among HIV-positive women in Kenya. Int J Cancer.

[20] Taghavi K., Moono M., Mwanahamuntu M., Roumet M., Limacher A., Kapesa H. (2024). Accuracy of screening tests for cervical precancer in women living with HIV in low-resource settings: a paired prospective study in Lusaka, Zambia. BMJ Oncol.

[21] Waxman A.G., Chelmow D., Darragh T.M., Lawson H., Moscicki AB. (2012). Revised terminology for cervical histopathology and its implications for management of high-grade squamous intraepithelial lesions of the cervix. Obstet Gynecol.

[22] Sabol I., Salakova M., Smahelova J., Pawlita M., Schmitt M., Gasperov N.M. (2008). Evaluation of different techniques for identification of human papillomavirus types of low prevalence. J Clin Microbiol.

[23] Schiffman M.H., Kiviat N.B., Burk R.D., Shah K.V., Daniel R.W., Lewis R. (1995). Accuracy and interlaboratory reliability of human papillomavirus DNA testing by hybrid capture. J Clin Microbiol.

[24] Clifford G.M., de Vuyst H., Tenet V., Plummer M., Tully S., Franceschi S. (2016). Effect of HIV infection on human papillomavirus types causing invasive cervical cancer in Africa. J Acquir Immune Defic Syndr.

[25] Pankam T., Kerr S.J., Teeratakulpisan N., Rodbamrung P., Wongkanya R., Keelawat S. (2017). Human papillomavirus in anal biopsy tissues and liquid-based cytology samples of HIV-positive and HIV-negative Thai men who have sex with men. Papillomavirus Res.

[26] Castro F.A., Koshiol J., Quint W., Wheeler C.M., Gillison M.L., Vaughan L.M. (2015). Detection of HPV DNA in paraffin-embedded cervical samples: a comparison of four genotyping methods. BMC Infect Dis.

[27] Bottari F., Passerini R., Renne G., Guerrieri M.E., Sandri M.T., Li A. (2021). Onclarity performance in human papillomavirus DNA detection in formalin-fixed paraffin-embedded cervical samples. J Low Genit Tract Dis.

[28] Kim G., Cho H., Lee D., Park S., Lee J., Wang H.Y. (2017). Comparison of FFPE histological versus LBP cytological samples for HPV detection and typing in cervical cancer. Exp Mol Pathol.

[29] Ye Z., Zhao Y., Chen M., Lu Q., Wang J., Cui X. (2024). Distribution and diagnostic value of single and multiple high-risk HPV infections in detection of cervical intraepithelial neoplasia: a retrospective multicenter study in China. J Med Virol.

[30] Chen S., Du Q., Yin J., Xu X., Chen W., Pan Q. (2025). HPV genotyping agreement between paired cervical cytological sample and biopsy across lesion severity and vaccination. Virol J.

