# Supplementary material for: Human papillomavirus detection in liquid-based cytology and cervical histology samples among women living with human immunodeficiency virus in Zambia
Source: IJID Reg. 2026 Aug 11;21:100973. doi: 10.1016/j.ijregi.2026.100973 (PMC13633901; doi:10.1016/j.ijregi.2026.100973)
Supplement: Supplementary file 1 [file mmc1.docx]

Supplementary Material

**Supplementary Figure 1.** Flow diagram showing the selection of study participants.

HPV, human papillomavirus.

**Supplementary Table 1.** Agreement of Group 2 and low-risk HPV genotypes in cytology and histology samples.

|  |  | **Cytology** | **Histology** | |  | **Overall** | | |
| --- | --- | --- | --- | --- | --- | --- | --- | --- |
|  | **HPV result** |  | **positive** | **negative** | **Total** | **Agreement**  **(%)** | **Positive agreement (%)** | **Kappa**  **(95% CI)** |
| **Group 2A** | HPV 68 | positive | 1 | 10 | 11 | 95.9 | 16.7 | 0.16 |
|  |  | negative | 0 | 231 | 231 |  |  | (-0.12, 0.44) |
|  |  |  | 1 | 241 | 242 |  |  |  |
| **Group 2B** | HPV 26 | positive | 1 | 2 | 3 | 97.6 | 33.3 | 0.33 |
|  |  | negative | 2 | 237 | 239 |  |  | (-0.17, 0.82) |
|  |  |  | 3 | 239 | 242 |  |  |  |
|  | HPV 53 | positive | 3 | 3 | 6 | 98.6 | 60.0 | 0.59 |
|  |  | negative | 1 | 235 | 236 |  |  | (0.23, 0.96) |
|  |  |  | 4 | 238 | 242 |  |  |  |
|  | HPV 66 | positive | 2 | 7 | 9 | 96.3 | 30.8 | 0.29 |
|  |  | negative | 2 | 231 | 233 |  |  | (-0.04 , 0.62) |
|  |  |  | 4 | 238 | 242 |  |  |  |
|  | HPV 69 | positive | 2 | 4 | 6 | 97.9 | 44.4 | 0.43 |
|  |  | negative | 1 | 235 | 236 |  |  | (0.02, 0.84) |
|  |  |  | 3 | 239 | 242 |  |  |  |
|  | HPV 73 | positive | 0 | 3 | 3 | 98.4 | 0 | -0.01 |
|  |  | negative | 1 | 238 | 239 |  |  | (-0.02, 0.01) |
|  |  |  | 1 | 241 | 242 |  |  |  |
|  | HPV 82 | positive | 1 | 2 | 3 | 97.6 | 33.3 | 0.33 |
|  |  | negative | 2 | 237 | 239 |  |  | (-0.17, 0.82) |
|  |  |  | 3 | 239 | 242 |  |  |  |
| **Low risk** | HPV 6 | positive | 0 | 5 | 5 | 94.2 | 0 | -0.03 |
|  |  | negative | 9 | 228 | 237 |  |  | (-0.05,-0.00) |
|  |  |  | 9 | 233 | 242 |  |  |  |
|  | HPV 11 | positive | 1 | 0 | 1 | 95.5 | 15.4 | 0.15 |
|  |  | negative | 11 | 230 | 241 |  |  | (-0.11, 0.41) |
|  |  |  | 12 | 230 | 242 |  |  |  |
|  | HPV 40 | positive | 0 | 5 | 5 | 97.1 | 0 | -0.01 |
|  |  | negative | 2 | 235 | 237 |  |  | (-0.03, - 0.01) |
|  |  |  | 2 | 240 | 242 |  |  |  |
|  | HPV 42 | positive | 5 | 4 | 9 | 97.5 | 62.5 | 0.61 |
|  |  | negative | 2 | 231 | 233 |  |  | (0.33 , 0.90) |
|  |  |  | 7 | 235 | 242 |  |  |  |
|  | HPV 43 | positive | 0 | 6 | 6 | 95.9 | 0 | -0.02 |
|  |  | negative | 4 | 232 | 236 |  |  | (-0.04, 0.00) |
|  |  |  | 4 | 238 | 242 |  |  |  |
|  | HPV 44 | positive | 0 | 2 | 2 | 98.7 | 0 | -0.01 |
|  |  | negative | 1 | 239 | 240 |  |  | (-0.02, 0.01) |
|  |  |  | 1 | 241 | 242 |  |  |  |
|  | HPV 54 | positive | 1 | 7 | 8 | 97.1 | 22.2 | 0.22 |
|  |  | negative | 0 | 234 | 234 |  |  | (-0.14, 0.57) |
|  |  |  | 1 | 241 | 242 |  |  |  |
|  | HPV 61 | positive | 0 | 4 | 4 | 97.5 | 0 | -0.01 |
|  |  | negative | 2 | 236 | 238 |  |  | (-0.03, 0.01) |
|  |  |  | 2 | 240 | 242 |  |  |  |
|  | HPV 70 | positive | 2 | 2 | 4 | 99.1 | 66.7 | 0.66 |
|  |  | negative | 0 | 238 | 238 |  |  | (0.23, 1.00) |
|  |  |  | 2 | 240 | 242 |  |  |  |

HPV – human papillomavirus; lrHPV –any low-risk HPV genotype (HPV 6, 11, 40, 42, 43, 44, 54, 61, and 70); Group 1 HPV – any IARC Group 1 carcinogenic genotypes (16, 18, 31, 33, 35, 39, 45, 51, 52, 56, 58, 59); Group 2 HPV – any IARC Group 2 genotypes (2A probably carcinogenic: 68; 2B possibly carcinogenic: 26, 53, 66, 69, 73, 82); CI – confidence interval.

**Supplementary Table 2:** Agreement of HPV genotypes in cervical cytology and histology samples among women who received precancer treatment since baseline (n = 46) and those who did not receive precancer treatment since baseline (n = 196).

|  | **Cytology** | **Histology** | |  | **Women with precancer treatment since baseline** | | |
| --- | --- | --- | --- | --- | --- | --- | --- |
| **HPV result** |  | Positive | Negative | Total | Overall agreement (%) | Positive agreement (%) | Kappa  (95% CI) |
| HPV 16 | Positive | 4 | 1 | 5 |  |  |  |
|  | Negative | 4 | 37 | 41 | 89.13 | 61.53 | 0.56 |
|  | Total | 8 | 38 | 46 |  |  | (0.22 - 0.90) |
| Any hrHPV | Positive | 13 | 6 | 19 |  |  |  |
| (all 19 genotypes) | Negative | 5 | 22 | 27 | 76.10 | 70.27 | 0.50 |
|  | Total | 18 | 28 | 46 |  |  | (0.25 - 0.76) |
| Any Group 1 | Positive | 9 | 6 | 31 |  |  |  |
| (12 genotypes) | Negative | 5 | 26 | 15 | 71.74 | 62.06 | 0.47 |
|  | Total | 14 | 32 | 46 |  |  | (0.17 - 0.72) |
| Any Group 2 | Positive | 2 | 3 | 5 |  |  |  |
| (7 genotypes) | Negative | 2 | 39 | 41 | 89.13 | 44.44 | 0.39 |
|  | Total | 4 | 42 | 46 |  |  | (-0.05 - 0.82) |
| Any lrHPV | Positive | 1 | 4 | 5 |  |  |  |
| (9 genotypes) | Negative | 6 | 35 | 41 | 78.26 | 16.67 | 0.05 |
|  | Total | 7 | 39 | 46 |  |  | (-0.26 - 0.35) |
|  | **Cytology** | **Histology** | |  | **Women without precancer treatment since baseline** | | |
| **HPV result** |  | Positive | Negative |  | Overall agreement (%) | Positive agreement (%) | Kappa (95% CI) |
| HPV 16 | Positive | 5 | 12 | 17 |  |  |  |
|  | Negative | 23 | 156 | 179 | 82.14 | 22.22 | 0.13 |
|  | Total | 28 | 168 | 196 |  |  | (-0.04 - 0.30) |
| Any hrHPV | Positive | 35 | 41 | 76 |  |  |  |
| (all 19 genotypes) | Negative | 30 | 90 | 120 | 63.78 | 49.64 | 0.22 |
|  | Total | 65 | 131 | 196 |  |  | (0.08 - 0.36) |
| Any Group 1 | Positive | 28 | 34 | 62 |  |  |  |
| (12 genotypes) | Negative | 29 | 105 | 134 | 62.76 | 47.06 | 0.18 |
|  | Total | 57 | 139 | 196 |  |  | (0.10 - 0.38) |
| Any Group 2 | Positive | 6 | 17 | 23 |  |  |  |
| (7 genotypes) | Negative | 5 | 168 | 173 | 88.78 | 35.29 | 0.30 |
|  | Total | 11 | 185 | 196 |  |  | (0.09 - 0.51) |
| Any lrHPV | Positive | 9 | 23 | 32 |  |  |  |
| (9 genotypes) | Negative | 17 | 147 | 164 | 79.59 | 31.03 | 0.192 |
|  | Total | 26 | 170 | 196 |  |  | (0.02 - 0.36) |

HPV – human papillomavirus; hrHPV – any high-risk HPV genotype; lrHPV –any low-risk HPV (HPV 6, 11, 40, 42, 43, 44, 54, 61, and 70); Group 1 HPV – any IARC Group 1 carcinogenic genotypes (16, 18, 31, 33, 35, 39, 45, 51, 52, 56, 58, 59); Group 2 HPV – any IARC Group 2 genotypes (2A probably carcinogenic: 68; 2B possibly carcinogenic: 26, 53, 66, 69, 73, 82); CI – confidence interval.
